# InGaAs Diodes for Terahertz Sensing—Effect of Molecular Beam Epitaxy Growth Conditions

**DOI:** 10.3390/s18113760

**Published:** 2018-11-03

**Authors:** Vilius Palenskis, Linas Minkevičius, Jonas Matukas, Domas Jokubauskis, Sandra Pralgauskaitė, Dalius Seliuta, Bronislovas Čechavičius, Renata Butkutė, Gintaras Valušis

**Affiliations:** 1 Institute of Applied Electrodynamics and Telecommunications, Physics Faculty, Vilnius University, Sauletekio ave. 3, 10257 Vilnius, Lithuania; vilius.palenskis@ff.vu.lt (V.P.); jonas.matukas@ff.vu.lt (J.M.); sandra.pralgauskaite@ff.vu.lt (S.P.); 2 Department of Optoelectronics, Center for Physical Sciences and Technology, Savanoriu ave. 231, 02300 Vilnius, Lithuania; linas.minkevicius@ftmc.lt (L.M.); dalius.seliuta@ftmc.lt (D.S.); bronislovas.cechavicius@ftmc.lt (B.Č.); renata.butkute@ftmc.lt (R.B.); gintaras.valusis@ftmc.lt (G.V.)

**Keywords:** terahertz sensing, terahertz imaging, InGaAs diodes, molecular beam epitaxy, low-frequency noise, spectral density

## Abstract

InGaAs-based bow-tie diodes for the terahertz (THz) range are found to be well suited for development of compact THz imaging systems. To further optimize design for sensitive and broadband THz detection, one of the major challenges remains: to understand the noise origin, influence of growth conditions and role of defects for device operation. We present a detailed study of photoreflectance, low-frequency noise characteristics and THz sensitivity of InGaAs bow-tie diodes. The diodes are fabricated from InGaAs wafers grown by molecular beam epitaxy (MBE) on semi-insulating InP substrate under different technological conditions. Photoreflectance spectra indicated the presence of strong built-in electric fields reaching up to 49 kV/cm. It was demonstrated that the spectral density of voltage fluctuations at room temperature was found to be proportional to 1/*f*, while at lower temperatures, 77–200 K, Lorentzian-type spectra dominate due to random telegraph signals caused by individual capture defects. Furthermore, varying bias voltage, we considered optimal conditions for device room temperature operation in the THz range with respect to signal-to-noise ratio. The THz detectors grown with beam equivalent pressure In/Ga ratio equal to 2.04 exhibit the minimal level of the low-frequency noise, while InGaAs layers grown with beam equivalent pressure In/Ga ratio equal to 2.06 are found to be well suited for fabrication of room temperature bow-tie THz detectors enabling sensitivity of 13 V/W and noise equivalent power (NEP) of 200 pW/√Hz at 0.6 THz due to strong built-in electric field effects.

## 1. Introduction

The InGaAs/InP material system is one of the key compounds for ultrafast optoelectronics applications [1]. It can also serve as a core element for other optoelectronics applications including infrared photosensors [2] and magnetic field sensors [3]. A particular importance of this material compound arises from the rapidly growing interests in its suitability for efficient THz emission excited by femtosecond laser pulses [4,5], THz photomixers [6,7,8], possible use in nanometric field effect transistors for electrically driven plasma-wave-related THz emission [9], and THz sensing [10].

The InGaAs/InP material system is employed in the development of planar bow-tie-shaped InGaAs diodes [11] which were found well-suited for direct [12], heterodyne [13] and spectroscopic imaging applications up to 2.52 THz [14,15]. The diode operates both due to nonuniform carrier heating and resistive mixing caused by applied external THz electric field inducing DC voltage over the terminals of the diode [16].

The diode exhibits low noise because its operation does not require application of the bias voltage. The choice of the InGaAs material is motivated due to its high electron mobility, 13,300 cm^2^/V·s at room temperature [11]; the detector sensitivity at room temperature is of about 6 V/W, whilst the relevant value in GaAs-based detectors is about 0.3 V/W [17,18]. The noise-equivalent power (NEP) of InGaAs diodes in direct and heterodyne modes is estimated to be about 4 nW/√Hz and 0.23 pW/Hz, respectively, for a local-oscillator power of 11 µW [13].

Despite the fact that InGaAs bow-tie diode displays the advantages of broadband room temperature operation, rather uncomplicated fabrication process and good reliability, its NEP values need to be improved in comparison, for instance, to those of field effect transistors fabricated using CMOS technology (gate length is 150 nm), where NEP amounts to 43 pW/√Hz [19]. Therefore, to improve design of InGaAs-based bow-tie THz detectors, one needs to get deeper insight into origin of defects or recombination centers that may have impact on InGaAs layer and InGaAs/InAs/InP material interface properties.

Investigation of noise characteristics is well known as a highly sensitive method to reveal physical processes in various structures and devices as well as to enable the reliability prediction [20,21,22]. On the other hand, the noise level of a detector is the restricting factor of the imaging system sensitivity [23]. Preliminary investigation has shown that the spectral density of current fluctuations in bow-tie diodes changes with frequency approximately as 1/*f* and allows us to predict that the origin of noise is a superposition of the capture and emission of charge carriers in defects of the structure [24].

In this article, we discuss effects of different technological conditions for the InGaAs devices fabricated from InGaAs layers grown by molecular beam epitaxy (MBE) on semi-insulating InP substrate. It was shown that structures exhibit presence of strong built-in electric fields reaching up to 49 kV/cm, which were derived from the photoreflectance spectra. It was demonstrated that the spectral density of voltage fluctuations at room temperature is found to be proportional to 1/*f*, while at lower temperatures, 77–200 K, Lorentzian-type spectra dominate due to random telegraph signals caused by individual capture defects. The presented results demonstrate how InGaAs layers grown at different beam equivalent pressure (BEP) In/Ga ratios affect the low-frequency noise level and the sensitivity of the THz detectors. We underline that THz detectors grown with BEP In/Ga ratio equal to 2.06 are found to be most sensitive at room temperature.

## 2. Grown Structures and Experimental Techniques

The InGaAs layers for detectors were grown using the SVT-A MBE reactor on semi-insulating InP:Fe substrates oriented in the (100) crystallographic plane. Before the growth procedure, the surface of InP:Fe substrate was cleaned from native oxide by annealing at temperature of 500 °C for 10 min. In order to prevent the loss of phosphorus on the surface of the substrate, the deoxidation was performed in flux at a pressure of about 5 × 10^−6^ Torr in-situ observing the reflection high-energy electron diffraction (RHEED) pattern. After the native oxide removal, 1–2 monolayer (ML)-thick InAs was deposited. The thickness of the InAs layer was designed in such a way that it forms a structurally strained interlayer between the substrate and InGaAs. Then, the InGaAs layer was grown in a temperature range from 500 °C to 515 °C varying growth rate from 300 nm/h to 600 nm/h. Growth rate was determined from RHEED intensity oscillations recorded for the specular beam. To optimize the growth conditions, the In/Ga BEP ratio varying from 2.04 to 2.08 was investigated.

The patterning of a 1.18-µm-height mesa structure was carried out by optical lithography and wet etching [13]. The electrical contacts for detectors were deposited by thermal evaporation of 20-nm-thick Ti and 180-nm-thick Au. Contact layers after deposition were ex-situ annealed at 400 °C temperature for 10 s in nitrogen ambience using a rapid thermal annealing (RTA) oven.

**Figure 1:**
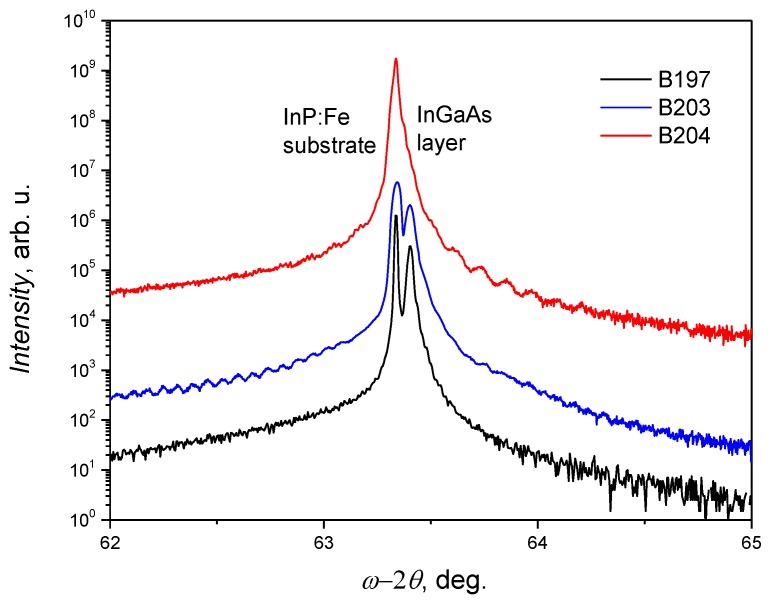
The rocking curves of (400) reflex of X-ray diffraction measured for detectors grown with different BEP In/Ga ratios: 2.04 (sample B197), 2.06 (sample B203), and 2.08 (sample B204).

**Figure 2:**
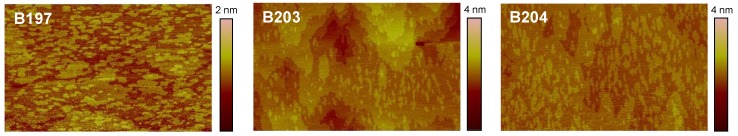
The surface morphology images of InGaAs epitaxial layers, grown using different BEP In/Ga ratios (2.04—B197, 2.06—B203, and 2.08—B204), obtained by atomic force microscopy. Scanned area is 1.5 × 0.7 m^2^.

The crystalline structure of In and Ga content in different InGaAs samples has been investigated by measuring high-resolution X-ray diffraction (HRXRD) ω-2θ scans of the (400) reflex using CuK_α1_ radiation. The results of XRD rocking curve measurements are presented in Figure 1. From these data, the lattice constant has been evaluated. The lattice constant varied from 5.8639 Å to 5.8687 Å for InGaAs layers grown using In/Ga ratios from 2.04 to 2.08, respectively, and then the In and Ga content was obtained. It was found that for InGaAs layers well matched to the InP:Fe substrate growth, the BEP ratio of In/Ga of about 2.08 is optimum. The growth mechanism, completion of atomic layers and the surface roughness have been investigated by atomic force microscopy (AFM). The surface images of InGaAs epitaxial layers are shown in Figure 2. AFM revealed that surface roughness of investigated InGaAs layers varied from 0.166 nm (B204) to 0.322 nm (B203). It was demonstrated that lattice matched to the InP substrate epitaxial InGaAs layer exhibits much smoother surface due to the layer-by-layer growth mode.

The photoluminescence (PL) measurements were carried out using the DPSS laser (532 nm) for an excitation with the intensity reaching ~600 W/cm^2^ and the 0.4-m monochromator. The PL was detected using a liquid nitrogen-cooled InGaAs point photodetector, whose signal was recorded using a lock-in amplifier when the laser radiation intensity had been modulated by a mechanical chopper set to 190 Hz modulation frequency.

The photoreflectance (PR) measurements were performed using a DPSS laser (532 nm) with modulation pump source chopped at 190 Hz. The pump beam intensity was kept below 5 mW cm^−2^.

Noise characteristics at forward and backward bias were measured within the frequency range from 10 Hz to 20 kHz varying lattice temperature from 77 K to 300 K. The experimental setup [24] is described in detail in Supplementary Materials.

Terahertz sensitivity was evaluated measuring responsivity of the InGaAs bow-tie detectors at frequency of 0.3 THz and 0.6 THz employing an electronic multiplier chain (Virginia Diodes, Inc. Charlottesville, VA, USA) as THz radiation source.

The AFM images (Figure 2) show the surface morphology obtained on GaInAs epitaxial layers, grown using different BEP In/Ga ratios (2.04—B197, 2.06—B203, and 2.08—B204). Scanned surface area is 1.5 × 0.7 µm^2^. The color scale of 4 nm resolution demonstrates surface roughness measured from peak (min) to peak (max). The roughness values measured on the surfaces of B197, B203 and B204 samples are presented in Table 1. The surface morphology of all investigated samples is very smooth and the roughness does not exceed 1 ML. Also, it is clearly seen from the obtained images for B197 and B203 samples (they are grown at the same temperature of about 500 °C but using different In/Ga ratios) that the atomic traces on the B203 image are wider and exhibit higher quality than B197. On the other hand, the contrast of colors on the B204 sample image is the lowest and shows the highest crystallinity.

**Figure 3:**
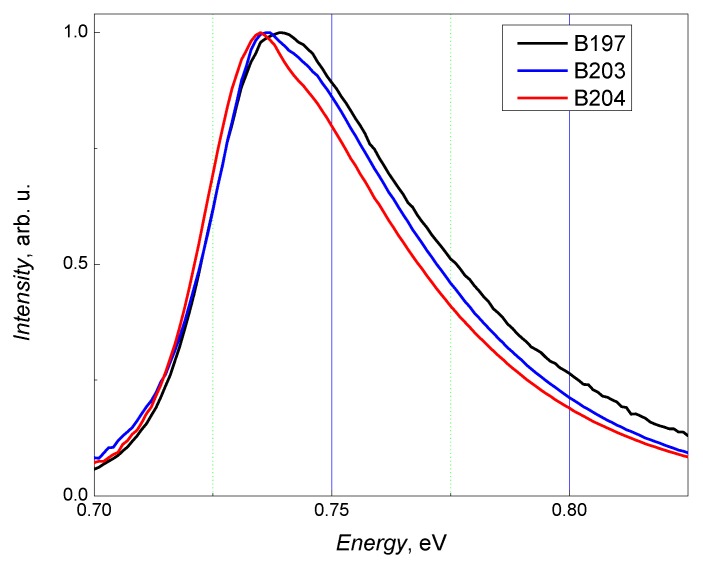
Room temperature photoluminescence spectra of three GaInAs samples: sample B197 (black line), B203 (blue line) and B204 (red line) grown using different BEP In/Ga ratios ranging from 2.04, 2.06 and 2.08, respectively, for B197, B203 and B204 at two substrate temperatures of about 500 °C (sample B197 and B203) and 510 °C (sample B204).

Figure 3 presents PL spectra measured at room temperature on three 540-nm-thick InGaAs layers grown in varying BEP ratios of In and Ga elements. It is seen that PL peak position is slightly shifted to the lower energies with increase of BEP ratio (In/Ga) from 2.04 to 2.08 (this ratio corresponds to the GaInAs composition lattice-matched to InP substrate). Although the full width at half maximum (FWHM) is less than 50 meV for all three layers, the B204 sample grown at substrate temperature of about 510 °C demonstrates the higher crystalline quality and the narrowest FWHM value (~40 meV).

**Figure 4:**
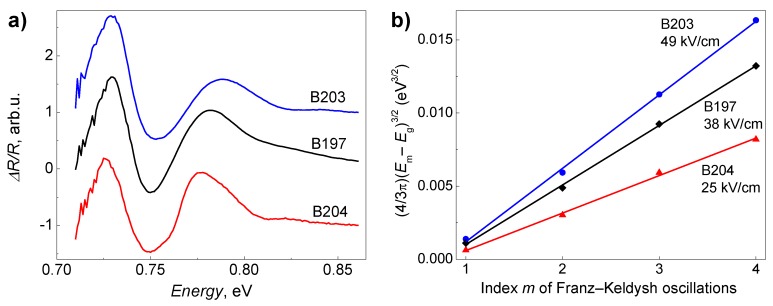
Photoreflectance spectra of InGaAs layers (**a**), each spectrum shifted vertically by 1 for clarity; and plots of the quantity (4/3π) (*E_m_* − *E_g_*)^3/2^ versus index *m* of Franz–Keldysh oscillations for epitaxial layers (**b**).

Room temperature PR spectra for the epitaxial InGaAs layers are shown in Figure 4. The spectra are dominated by characteristic oscillations. These PR features are associated with Franz–Keldysh oscillations (FKOs) indicating the existence of the internal electric field in the samples. By analysing FKOs above the band edge, it is possible to estimate the strength of the electric field. In the analysis of the electric field strength, the extremes of the FKOs are given by [25]

(1) $$m\pi =\frac{4}{3}{\left(\frac{E_{m}-E_{g}}{\hbar \theta }\right)}^{3/2}+\varphi ,$$

where *m* is the index of the *m*-th extremum, *E_m_* is the photon energy of the *m*-th extremum, *E_g_* is the band gap of InGaAs, *φ* is a phase factor. Electro-optic energy ℏ*θ* is given by (ℏ*θ*)^3^ = *e*^2^*F*^2^ℏ^2^/2*μ*, and depends on electric field *F,* and charge carrier reduced mass *μ*. Figure 4b depicts the quantity (4/3π) (*E_m_* − *E_g_*)^3/2^ plotted versus Franz–Keldysh oscillations index *m*. As it is seen, the function is linear (solid lines show linear fit to Equation (1)), and from the slope it allows one to evaluate the electro-optic energy, and, hence, to determine the built-in electric field strength in the structure [26]. Evaluation indicates that the strongest electric field—reaching 49 kV/cm—contains the structure B203. Technological characteristics of the investigated samples are summarized in Table 1.

## 3. Low-Frequency Noise Measurement Results and Discussion

### 3.1. Investigation of the Resistivity of the Detectors

**Figure 5:**
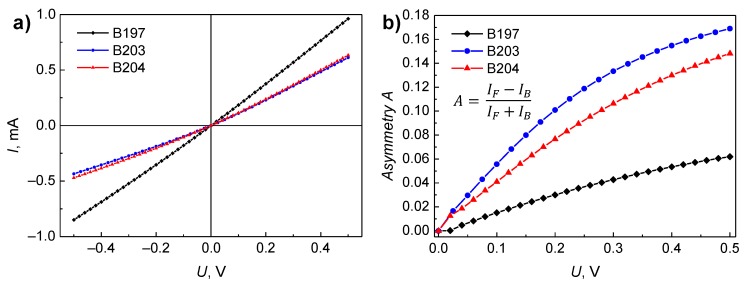
The current–voltage characteristics of InGaAs bow-tie detectors at room temperature (298 K) (**a**) and evaluated asymmetry (**b**), where *I_F_* and *I_B_* are forward and backward current values, respectively, at selected voltages.

**Figure 6:**
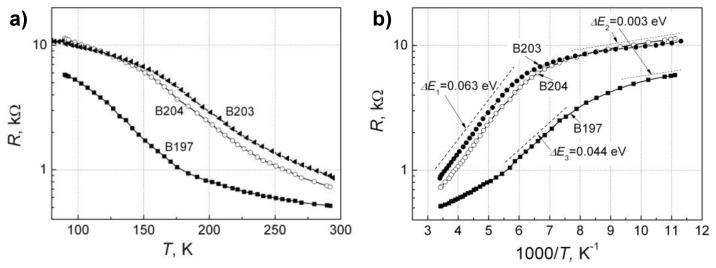
Temperature dependences of the resistance of the investigated bow-tie detectors B197, B203 and B204 in the linear part of *I–V* characteristics (**a**). The activated temperature dependences of the resistances of bow-tie detectors as a function of 1000/*T* (**b**).

Typical current–voltage characteristics of the investigated bow-tie THz detectors at room temperature are represented in Figure 5, and the dependence of resistance on temperature at low biases is shown in Figure 6. As it is seen from Figure 5, detector’s B197 resistance is smaller than the other detectors at room temperature. Besides, the asymmetry of *I*–*U* characteristics for diode B203 and B204 is higher than those for B197 (Figure 5b). It is worth noting that at temperatures *T* > 200 K, the resistances slowly decrease with increase in temperature (Figure 6a), while at *T* < 200 K the resistances increase steeper with the decrease in temperature, especially characteristic for the detector B197. Such resistance changes with temperature can be described in terms of the activation energy (Figure 6b). The decrease of the activation energy ∆*E* with temperature observed for the sample B204 can be explained by the electron hopping mechanism in the impurity band in the energy gap of the semiconductor.

According to the Mott model, the charge carriers are transported via thermally activated tunneling of electrons between the localized states which are randomly distributed in energy and position [27,28]. Then, the electrical conductivity can be described as

(2) $$\sigma \sim exp\left(-{\left(\frac{T_{0}}{T}\right)}^{-\frac{1}{4}}\right),$$

where

(3) $$T_{0}=\frac{\lambda \alpha^{3}}{\left(N\left(E_{F}\right)k\right)},$$

*λ* labels the dimensionless constant, *α*^−1^ is the localization length of the wave function for localized states, *N*(*E*_F_) denotes the localized state density at the Fermi level, and *k* is the Boltzmann’s constant. *T*_0_ is the quantity with temperature units, which determines the rate of conductivity change with temperature.

### 3.2. Investigation of the Low-Frequency Noise of the Detectors

**Figure 7:**
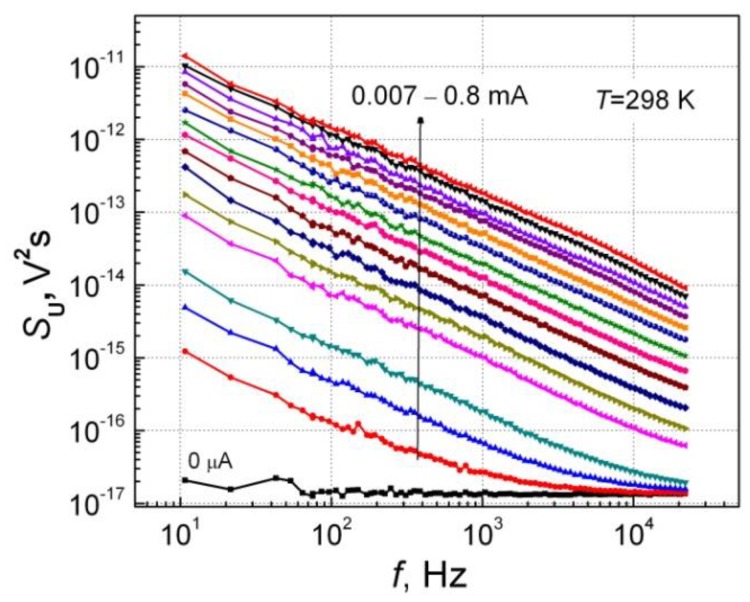
Voltage fluctuation spectra at different forward currents at room temperature for sample B204. The dependences for other samples are very similar.

Typical low-frequency noise spectra of the investigated InGaAs bow-tie detector B204 at different forward bias at room temperature (298 K) are presented in Figure 7 (the noise spectra for other samples as well as backward current direction at room temperature are almost the same as for the forward current direction, and therefore are not given here). The 1/*f*-type-voltage noise spectra show that the observed fluctuations are caused by the superposition of the charge carrier capture and emission processes in defects in the InGaAs layer and its interfaces [29,30,31].

A detailed description of the generation of noise with spectrum 1/*f*-type is presented in [29,30]. The low-frequency noise level caused by the charge carrier capture in localized states of defects in homogeneous semiconductors can be described as [31]

*S_U_*(*f*)/U^2^ = *S_R_*(*f*)/*R*^2^ = [0.16*K*(1 + *β*)^2^]/(*N*^2^*f*), (4)

where *S_R_*(*f*) is power spectral density of the resistance fluctuation Δ*R*(*t*); *K ≥* 1 is the average number of relaxators (localized capture centers) in the sample with arbitrarily distributed relaxation times in every double octave of relaxation times; *N* is the total number of free carriers in the sample; and *β* is the parameter that describes the contribution of the mobility of the charge carriers’ fluctuation to the total resistance fluctuation due to the free charge carrier capture process.

**Figure 8:**
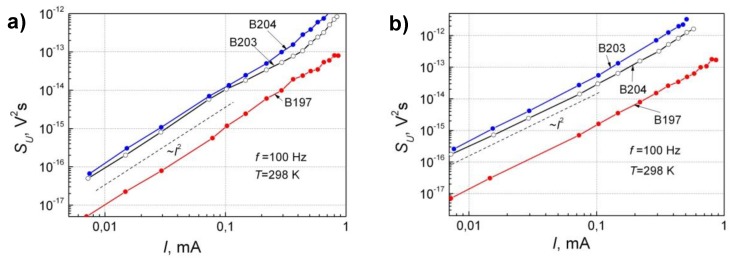
Spectral density voltage fluctuation dependence on bias current for forward (**a**) and backward current directions (**b**) at room temperature and frequency *f* = 100 Hz.

The dependences of the spectral density of voltage fluctuations on forward and backward bias current are shown in Figure 8. From the comparison of Figure 5 and Figure 8, it is seen that in the linear current–voltage characteristics range (*U* < 0.2 V), the spectral density of voltage fluctuations can be expressed by detector’s resistance, *R*, and fluctuation spectral density *S_R_*(*f*) [18]:

(5) $$S_{U}\left(f\right)=U^{2}\frac{S_{R}\left(f\right)}{R^{2}}=I^{2}S_{R}\left(f\right).$$

The 1/*f* noise level in the backward current direction is slightly higher than that in the forward one, and this correlates with the fact that the differential resistance in the backward current direction is larger than that in the forward direction. The low-frequency noise level at room temperature for the detector B197 is about an order of magnitude lower than that for other samples, because the differential resistance at room temperature for this detector is also about two-times smaller (Figure 6). This result is related to the smaller In/Ga BEP ratio during growth of sample B197 and its small surface roughness (rms). From the point of view of the low-frequency noise level, the optimal BEP In/Ga ratio is 2.04. According to Equation (4), the parameter *K* describing the localized defect density for this sample is about an order of magnitude smaller than that for other diodes.

**Figure 9:**
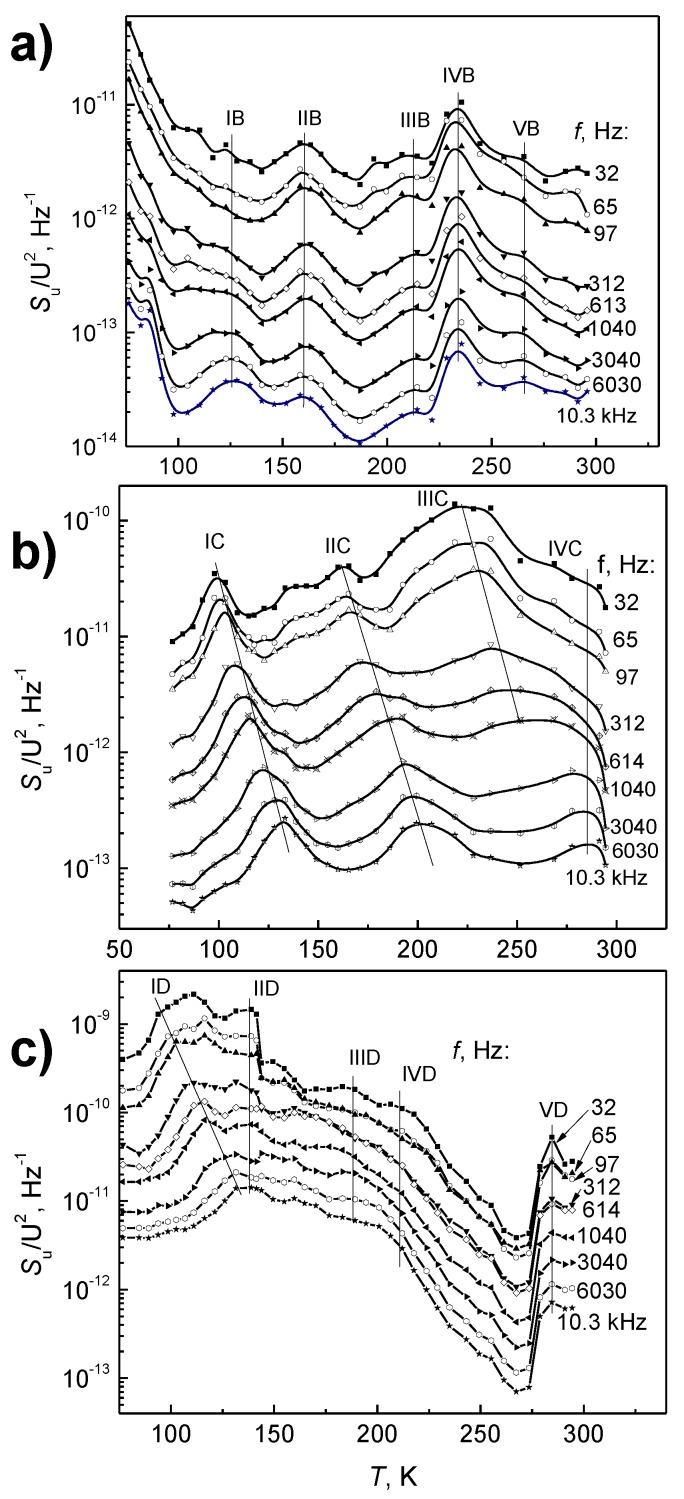
The normalized noise level dependences on temperature at different frequencies (**a**) for detector B197; (**b**) for B203; (**c**) for B204. The lines are included as a guide for the eye and illustrate changes of the noise peak groups.

The low-frequency noise level dependences on temperature at different frequencies for the investigated detectors are presented in Figure 9. The noise peaks appear at particular temperatures due to the random telegraph signals (RTS) observed, when the Fermi energy level coincides with the energy of the defect capture center. The low-frequency noise is consistent with the model of individual fluctuators (capture centers) affecting the resistance locally over a small area, independent of the full area of the device [32]. The power spectrum of the resistance fluctuations due to the defect *k* can be written as [32]:

(6) $$S_{Rk}\left(f\right)=\frac{4\langle \Delta R_{k}^{2}\rangle }{\left(\langle \tau_{e}\rangle +\langle \tau_{c}\rangle \right)\left[{\left(\frac{1}{\tau_{eff}}\right)}^{2}+{\left(2\pi f\right)}^{2}\right]},$$

where

(7) $$\frac{1}{\tau_{eff}}=\frac{1}{\langle \tau_{e}\rangle }+\frac{1}{\langle \tau_{c}\rangle };$$

$\langle \tau_{e}\rangle$ is the average time until the electron is emitted from the *k* defect energy level, and $\langle \tau_{c}\rangle$ is the average time until the electron is captured by the defect level.

The local resistance fluctuations cause the voltage fluctuations (Figure 9), when the DC current flows through the device, and the spectral density of voltage fluctuations due to all active traps *N*_tr_ in the device can be described as

(8) $$S_{U}=4I^{2}\sum_{k=1}^{N_{tr}}\langle \Delta R_{k}^{2}\rangle \frac{\tau_{eff}}{\langle \tau_{e}\rangle +\langle \tau_{c}\rangle }\cdot \frac{\tau_{eff}}{1+{\left(2\pi \tau_{eff}\right)}^{2}},$$

where the summation is performed over all traps (*k* = 1, 2, …, *N_tr_*) contained in the InGaAs layers and their interfaces. The relative resistance *R* changes $\Delta R_{k}/R\sim 1/A$, where *A* is the InAs surface area, and for a constant trap density $N_{tr}\sim A$. If the total number of traps *N_tr_* is sufficiently large to ensure the wide distribution of the time constant, and if $\langle \Delta R_{k}^{2}\rangle$ value for every *k* defect is about the same order, then one can obtain the 1/*f*-type noise spectrum.

In the case of dot centers, which have the same energy level, one would expect irregular RTS signals, and the noise spectra with peaks, as it is shown in Figure 9. These noise spectra can be fitted with a few Lorentzian-type spectra (Equation (8)) with a small background of 1/*f* noise component. The Equation (8) reaches the maximal value when the Fermi level coincides with the defect localization energy in the bandgap. In this case, $\langle \tau_{ke}\rangle =\langle \tau_{kc}\rangle$. From these peaks, one can evaluate the effective relaxation times (Equation (7)), and their changes with temperature because every peak fulfils the condition: $2\pi f_{0k}\tau_{k\;eff}=1$.

In order to highlight the effective relaxation time changes, it is convenient to represent the obtained experimental results at different fixed frequencies, as shown in Figure 9. As it is seen from Figure 9a, the noise peak position for the detector B197 is practically independent of temperature. This happens at a specific temperature when the Fermi energy level is located in a continuum of defect energies, which causes a very wide distribution of relaxation times. In the case of the sample B197 (Figure 9a) at temperatures of 127 K, 160 K, 213 K, 234 K and 265 K, the relaxation time distribution ranges exceed the interval from 15 μs to 5 ms. As it is seen, other samples, B203 and B204 (Figure 9b,c), express stronger dependences on temperature in comparison to B197.

**Figure 10:**
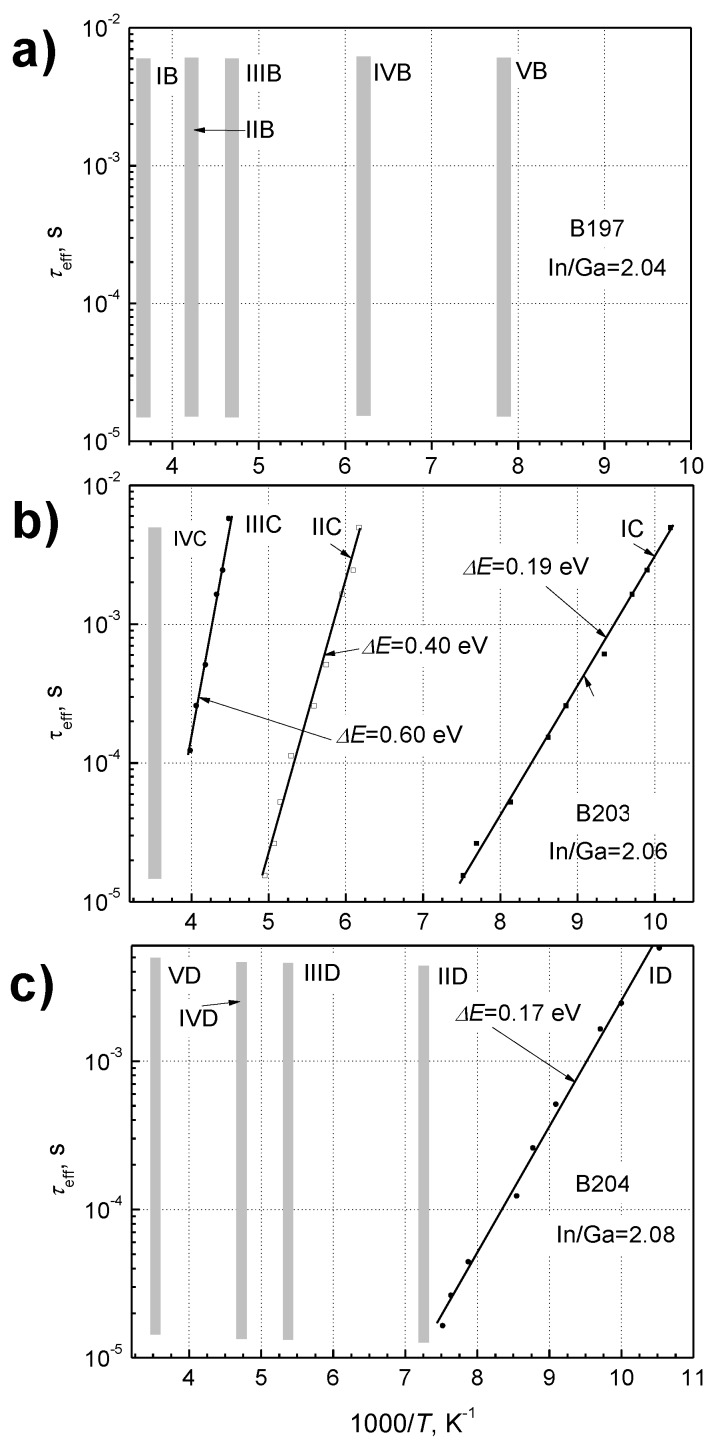
The relaxation time dependences on temperature for the investigated InGaAs bow-tie detectors with different In/Ga ratios: (**a**) for B197; (**b**) for B203; (**c**) for B204. Vertical columns represent the interval of the distribution of relaxation times at definite temperatures, calculated from the Figure 9. The evaluated activation energies are indicated near the relevant curves.

The evaluated relaxation times for every group of peaks of the investigated detectors are shown in Figure 10.

## 4. Terahertz Sensitivity Measurement Results and Discussion

**Figure 11:**
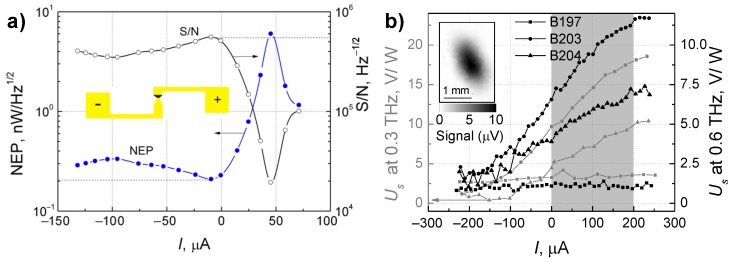
The noise equivalent power (NEP) at 0.6 THz (**a**) and voltage sensitivity (**b**) of the InGaAs diode detector with different In/Ga ratios at 0.3 THz and 0.6 THz frequency with modulation frequency of 1 kHz. Inset in the panel (**a**) shows the schematic design of the InGaAs bow-tie diode. Inset in the panel (**b**) indicates the raster scan of the 0.6 THz beam profile at the focal plane obtained with detector B203. Shadowed area in panel (**b**) indicates optimal working regime of the detector.

The bow-tie diodes were fabricated using lithography and wet etching technologies following the design of the bow-tie sensors described in [13]. The responsivity measurements of the InGaAs bow-tie detectors have been performed by 30 mW power radiation at frequency of 0.3 THz and 0.8 mW at 0.6 THz at modulation frequency of 1 kHz. The device position in the THz beam was set by raster scanning of the incident beam (Figure 11b) at the focal plane. The load resistance was set to 2.4 kΩ at 0.3 THz and 4.3 kΩ at 0.6 THz, respectively. The DC current was changed from –0.3 mA to 0.3 mA, where positive voltage was connected to the metalized taper part of the antenna. The results of the experimental investigation are presented in Figure 11.

The NEP of the B203 sample as a function of current (panel (a) of Figure 11) shows weak dependence at negative bias, and the minimal value of around 200 pW/√Hz is reached when the current approaches zero value. With the increase of forward bias the NEP value reaches the maximal value of 6 nW/√Hz at 5 µA. It is worth noting that the minimal NEP value obtained here—around 200 pW/√Hz—is much smaller than that reported earlier—4 nW/√Hz [13]—due to the more optimal growth conditions.

Figure 11b depicts the sensitivity as a function of current for all types of the studied samples at frequencies of 0.3 THz and 0.6 THz. At is seen, sensitivity of InGaAs bow-tie diodes fabricated from the wafers B203 and B204 increases while raising the current and reaches 17.5 V/W and 10 V/W at 0.3 THz and 12.5 V/W and 7 V/W at 0.6 THz, respectively, at the bias current of 0.2 mA. Higher voltage sensitivity of the detector B203 is caused by the larger asymmetry in the *I–V* curves and stronger built-in electric fields in comparison to other studied samples. Raster scan of THz beam profile at 0.6 THz (inset in panel (b) of Figure 11) illustrates the suitability of the diode for THz imaging aims.

Hence, operation parameters of the InGaAs-based bow-tie diodes can be optimized by varying conditions of the MBE growth. If the In/Ga BEP ratio is kept equal to 2.04, it allows one to reduce the low-frequency noise into the lowest level among the studied structures. The increase of the In/Ga BEP ratio to 2.06 enables one to increase the internal electric fields in the structure up to 49 kV/cm, making such wafers well suited for fabrication of sensitive—13 V/W and NEP of 200 pW/√Hz at 0.6 THz—room temperature bow-tie THz detectors and their further implementation in compact THz imaging systems [33]. The In/Ga BEP ratio set to 2.08 permits one to achieve the highest crystallinity; however, the internal electric fields—up to 25 kV/cm—seem to be not strong enough to provide maximal sensitivity values in the investigated diodes.

## 5. Conclusions

Noise characteristics of InGaAs bow-tie diodes are investigated over the temperatures from 77 K to 300 K. The voltage fluctuation spectral density varies proportionally to 1/*f* at room temperature, and Lorentzian-type noise spectra appear at lower temperatures. The origin of the fluctuations in the investigated bow-tie diodes is related to the defects caused by charge carrier capture centers involved in generation and recombination processes with characteristic times from a few microseconds to tens of milliseconds. The estimated activation energies of these centers are found to be in the range from 0.15 eV to 0.36 eV. It is supposed that they are associated with the presence of the interface defects close to the InAs layer located between the InGaAs layer and InP substrate. The THz detectors grown with beam equivalent pressure In/Ga ratio equal to 2.04 exhibit the minimal level of the low-frequency noise, while InGaAs layers grown with beam equivalent pressure In/Ga ratio equal to 2.06 are found to be well suited for fabrication of room temperature bow-tie THz detectors enabling sensitivity of 13 V/W and NEP of 200 pW/√Hz at 0.6 THz due to strong built-in electric field effects.

## Figures and Tables

**Table 1:** Technological conditions and parameters of characterization of investigated bow-tie detectors: BEP—beam equivalent pressure; ∆*2θ*—misalignment between the (400) reflexes of InP substrate and InGaAs epilayer; content of In in percent; *d*—thickness of InGaAs, nm; *rms*—surface roughness, nm; *R*—resistance, Ω; *E*—emission energy, eV.

Samples	B197	B203	B204
BEP ratio In/Ga	2.04	2.06	2.08
∆2*θ*, deg.	−0.07	−0.064	0
In, %	47.0	52.5	53.2
InGaAs layer thickness, *d*, nm	540	540	540
rms, nm	0.167	0.322	0.166
*R*, Ω	470–500	850–900	770–780
*E*, eV	0.739	0.736	0.735

## Supplementary Materials

The following are available online at http://www.mdpi.com/1424-8220/18/11/3760/s1. Figure S1: The noise measurement circuit: S is the investigated bow-tie detector; *R*_L_ denotes the load resistor; *R*_ref_ is the reference resistor; LNA stands for the low-noise amplifier; ADC labels the analog-to-digital converter (National InstrumentsTM PCI 6115 board); PC is the personal computer; Osc. shows the oscilloscope; Cr. indicates the cryostat (DN 7704); and V denotes the voltmeter.
