# Supplementary material for: InGaAs Diodes for Terahertz Sensing—Effect of Molecular Beam Epitaxy Growth Conditions
Source: Sensors (Basel). 2018 Nov 3;18(11):3760. doi: 10.3390/s18113760 (PMC6264118; doi:10.3390/s18113760)
Supplement: Supplementary file 1 [file sensors-18-03760-s001.pdf]

Supplementary materials

Experimental set-up for low-frequency noise measurements

Noise characteristics were measured in frequency range from 10 Hz to 20 kHz and lattice temperature ranging from 77 K to 300 K at forward and backward bias. The measuring system comprises of low-noise amplifier, filter system and analog-to-digital converter (National Instruments™ PCI 6115 board) (Fig. 1) [1]. The voltage noise measurements were performed in a constant current mode (the load resistance was at least 30-50 times higher than the resistance of the sample).

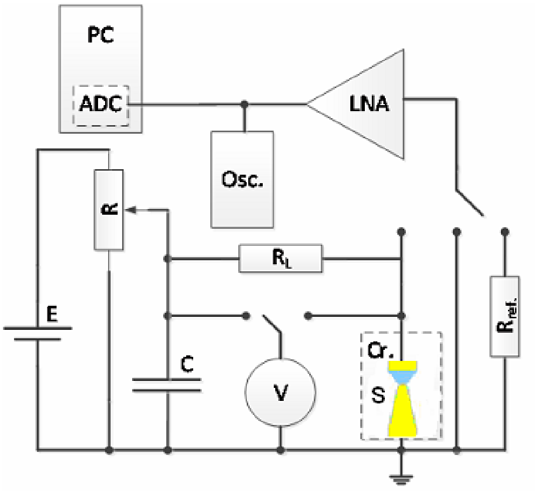

Figure 1. The noise measurement circuit: S is the investigated bow-tie detector; R_L denotes the load resistor; R_{ref} is the reference resistor; LNA stands for the low-noise amplifier; ADC labels the analog-to-digital converter (National InstrumentsTM PCI 6115 board); PC is the personal computer; Osc. shows the oscilloscope; Cr. indicates the cryostat (DN 7704); and V denotes the voltmeter.

The noise signal was processed with a computer-based fast Fourier transform signal analyzer. The voltage fluctuation spectral density was evaluated by comparison with thermal noise of the reference resistor [2] as follows:

$$S_U = \frac{<V^2> - <V_s^2>}{<V_{ref}^2> - <V_s^2>} 4kT_0 R_{ref} \qquad (1)$$

where $<V^2>$, $<V_s^2>$ and $<V_{ref}^2>$ are respectively the sample, measurement system including sample thermal noise, and the reference resistor thermal noise variances in the narrow frequency band Δf ; T_0 is the absolute temperature of the reference resistor; k is the Boltzmann's constant. The noise measurements were performed in a specially shielded room (Faraday cage) in order to avoid the interfering effects from electrical network and communication systems.

[1] Minkevičius, L.; Ragauskas, M.; Matukas, J.; Palenskis, V.; Pralgauskaitė, S.; Seliuta, D.; Kašalynas, I.; Valušis, G. InGaAs Bow-tie Diodes for Terahertz Imaging: Low Frequency Noise Characterization. Proc. SPIE Terahertz Emitters, Receivers, and Applications III, San Diego, CA, USA, 12-13 Aug 2012, Eds. M. Razeghi, A. N. Baranov, H. O. Everitt, J. M. Zavada; T. Manzur, **2012**, *8496*, 849612, DOI. 10.1117/12.929501.

[2] Palenskis, V.; Matukas, J.; Maknys, K.; Stadalnikas, A. Flicker noise generation mechanism and its relation to the defectiveness of materials and electronic devices, Proc. of Int. NODITO Workshop, 1995, 143-150.
